# Chondrosarcoma of Sternal Origin: A Rare Case

**DOI:** 10.7759/cureus.40393

**Published:** 2023-06-13

**Authors:** Swaragandha S Jadhav, Avinash P Dhok, Kajal R Mitra, Prashant M Onkar, Suresh V Phatak

**Affiliations:** 1 Department of Radiodiagnosis, NKP Salve Institute of Medical Sciences and Research Center, Nagpur, IND

**Keywords:** case report, tumor, malignancy, sternum, chondrosarcoma

## Abstract

Chondrosarcoma of sternal origin is extremely rare. Here, we present the case of a 59-year-old male who presented with gradually increasing swelling and pain over the left sternoclavicular joint which was diagnosed as chondrosarcoma of sternal origin. We also present a review of the relevant literature.

## Introduction

Chondrosarcoma of sternal origin is extremely rare. Its incidence is estimated to be only 15% of all chondrosarcomas reported worldwide. The annual predicted incidence of chest wall chondrosarcoma is 60 cases, with sternal origin accounting for 2% of these cases [1,2].

Soft-tissue sarcomas are mesenchymal-derived tumors that affect muscles, endothelium, cartilage, and supporting tissues but not the blood vessels or the reticuloendothelial system. Less than 1% of all neoplasias are primary malignant tumors of the thoracic wall, which can include a wide range of soft tissue and bone abnormalities [3].

Based on etiology, it can be categorized as primary if the tumor grows from nothing or secondary if it results from single or multiple existing benign osteochondromas and enchondromas. Osteochondromas experience secondary chondrosarcoma in around 80% of instances, with malignant transformation occurring slightly more frequently in solitary exostoses than in multiple exostoses. Isocitrate dehydrogenase 1 and 2 genes are one of the most frequent point mutations. Isocitrate dehydrogenase is a metabolic enzyme that facilitates the conversion of isocitrate to alpha-ketoglutarate through oxidative decarboxylation [4].

Neoplastic involvement of the sternum is extremely rare and considered malignant unless proven otherwise. Computed tomography (CT) with contrast or magnetic resonance imaging (MRI) with histopathological correlation is crucial in confirming the diagnosis of chondrosarcoma, and the treatment is surgery. The prognosis is poor for the axial skeleton. Complete resection leads to acceptable long-term survival for patients with chondrosarcoma.

## Case presentation

A 59-year-old male presented with complaints of swelling and pain over the left sternoclavicular joint for seven months which was gradually increasing in size. The patient reported no history of fever or trauma. On general examination, the patient was conscious and well-oriented. Systemic examination was within normal limits.

On local examination, a relatively well-defined swelling measuring approximately 4.4 × 4.2 cm was noted at the sternal area. It was hard in consistency and immobile. There was no evidence of a rise in local temperature. Moreover, there was no evidence of dilated veins. No movement was noted with deglutition.

The patient was advised a chest X-ray. A relatively well-defined lytic lesion was noted over the sternal region and a possible diagnosis of a tumor of skeletal origin was made. The patient was advised to undergo a CT of the chest and an MRI of the thorax.

On CT of the thorax, a relatively well-defined hypodense lesion measuring 4.1 × 4.6 × 4.7 cm (transverse × craniocaudal × anteroposterior) was noted over the sternal surface. It showed a chondroid matrix with ring and arch-like calcification.

**Figure 1:**
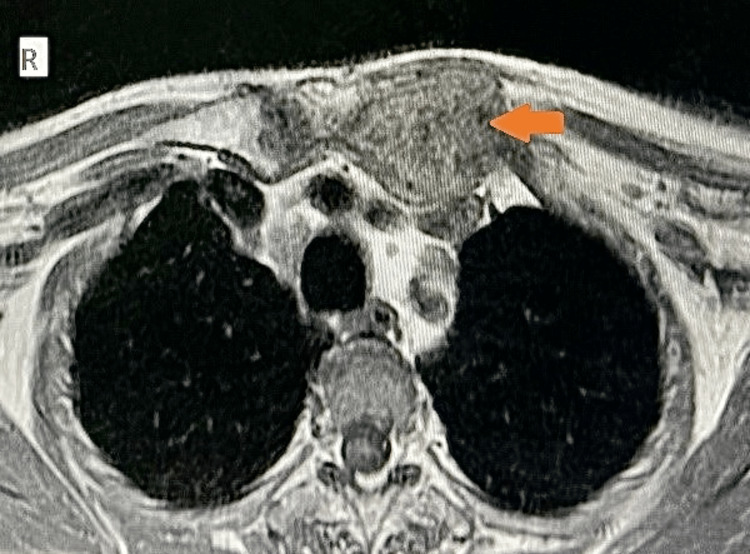
Plain MRI of the thorax axial T1-weighted image showing a relatively well-defined, altered signal intensity, heterogeneous, predominantly isointense lesion at the left sternoclavicular joint, juxta-cortical in a location involving the manubrium sterni and the medial end of the clavicle with adjacent soft-tissue components (arrow).

**Figure 2:**
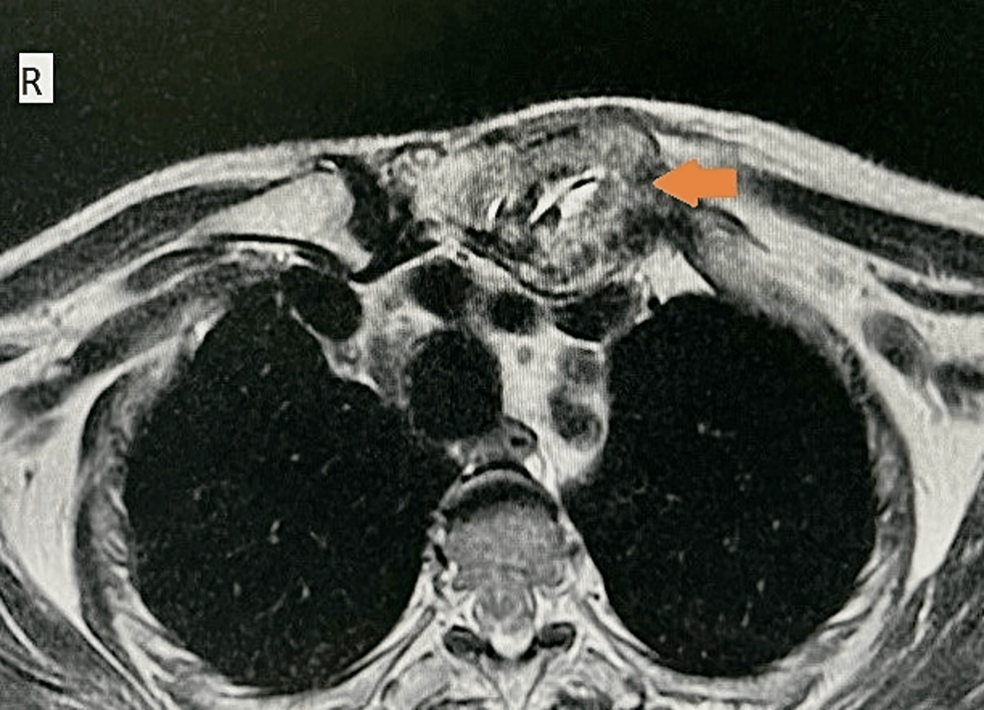
On the axial T2-weighted image, the lesion appears heterogeneous and predominantly hyperintense (arrow).

On MRI of the thorax, a relatively well-defined altered signal intensity lesion measuring 4.1 × 4.6 × 4.7cm (transverse × craniocaudal × anteroposterior) was noted at the left sternoclavicular region at the juxta-cortical location involving the manubrium, the proximal body of sternum, medial end of the clavicle, and the first rib on the left side causing cortical bony destruction. The lesion was noted to be involving the adjacent soft tissue component, that is, the involvement of the adjacent pectoralis major muscle with maintained fat planes with a prevascular compartment of the anterior mediastinum. It appeared heterogeneous and predominantly isointense on the T1-weighted image (WI) (Figure 1), and heterogeneous and predominantly hyperintense on the T2WI (Figure 2).

**Figure 3:**
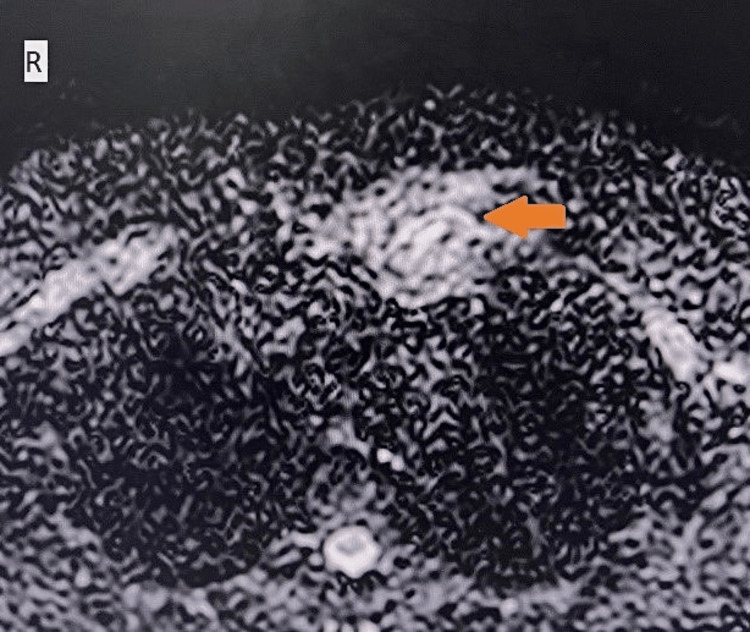
On diffusion-weighted imaging, the lesion shows areas of restriction (arrow).

On diffusion-weighted imaging (DWI), evidence of restriction was noted (Figure 3).

**Figure 4:**
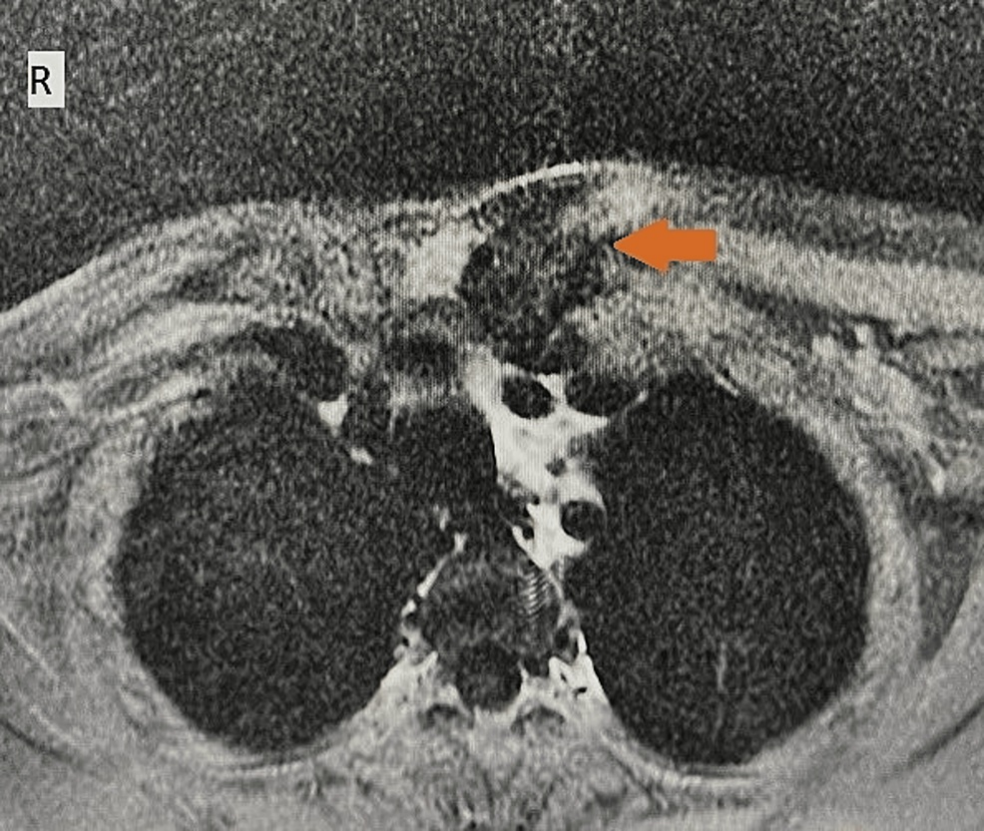
On the axial T1-weighted post-contrast image, the lesion shows heterogeneous enhancement (arrow).

On the post-contrast study, the lesion showed heterogeneous enhancement with few non-enhancing necrotic areas within (Figure 4).

The above-mentioned imaging features were highly suggestive of chondrosarcoma. A CT-guided biopsy was done and the result was grade I chondrosarcoma, with the presence of abundant extracellular hyaline matrix and scant cellularity. The patient underwent surgery. Subsequent follow-up visits were unremarkable.

## Discussion

Chondrosarcoma of sternal origin is an extremely rare tumor of soft mesenchymal origin. It occurs predominantly in males aged less than 20 years. It is an aggressive tumor, which in this case grew to invade the pleura and adjacent muscles. The clinical manifestation is a palpable mass that is rarely painful, causing respiratory failure and hemothorax. Although chondrosarcomas of sternal origin grow slowly, if not treated timely, they can undergo metastasis [2,3].

Soft-tissue sarcomas are lobulated, firm masses in gross appearance. Macroscopically, they are usually grayish-pink in color. They may also show hemorrhagic, necrotic, or calcific components within. On microscopy, these characteristically comprise small-to-medium-sized round cells with poor differentiation [4].

For patients with chondrosarcoma to receive successful therapy and have a better prognosis, early diagnosis is essential. Due to its varied clinical presentation, nonspecific symptoms, heterogeneous appearance on imaging examinations, and fluctuating biological behavior, the diagnosis can be difficult. Therefore, a combination of these factors must be taken into account to choose the best course of action. Differentiating between aggressive and more benign lesions is one of the most important variables. Larger size, localization in the pelvis and proximal regions of the limbs, the existence of multiple medullary lesions, and incidence in individuals aged over 50 years are specific characteristics of the lesions that may indicate a higher risk of malignancy [4].

Imaging diagnosis is based on multiple factors, including patient age, the pattern of bone destruction, and matrix mineralization, all of which may confirm the diagnosis in certain cases [2].

CT and MRI are the investigations of choice. CT helps diagnose calcification whereas MRI helps diagnose its soft-tissue extension [3]. MRI has greater soft-tissue contrast than other imaging methods. As a result, it is suitable for visualizing soft-tissue extension of chondrosarcoma. This method is also suitable for assessing bone marrow involvement. On T1-weighted images, marrow replacement caused by the lesion would appear as low/intermediate signal intensity patches. MRI also makes it possible to determine cortical damage, imprisoned fat, and peritumor edema. Before surgery, it is usually used to assess the lesion. When combined with radiographic techniques, it may be more effective in differentiating it from other lesions. Histopathological correlation is confirmative [4]. The choice of treatment is surgical, and it is resected with a normal tissue margin of 2-6 cm [3].

The growth and local relapse of thoracic wall chondrosarcomas is usually slow. Late metastases occur if untreated. The biggest factor affecting survival is whether the primary neoplasia is completely under control. A large excision must be performed during the initial procedure to stop local recurrence [3].

Other sarcomas such as synovial sarcomas, osteosarcomas of extraskeletal origin, and malignant fibrous histiocytomas are key differential diagnoses that need to be ruled out. Myositis ossificans is a benign differential that also needs to be considered [2].

## Conclusions

Chondrosarcoma of sternal origin is an uncommon tumor of the chest wall. It is diagnosed accurately by contrast-enhanced CT or MRI. Surgery is the mainstay of treatment.
